# Neutralization techniques and academic dishonesty among Italian university students

**DOI:** 10.3389/fpsyg.2026.1766612

**Published:** 2026-04-20

**Authors:** Emma Flutti, Guido Travaini, Paolo Roma, Martino Ongis

**Affiliations:** 1 Department of Human Neurosciences, Sapienza University of Rome, Rome, Italy; 2 Faculty of Medicine, University of Vita-Salute San Raffaele, Milan, Italy; 3 Department of Psychology, Franklin University Switzerland, Lugano, Switzerland

**Keywords:** academic dishonesty, higher education, moral reasoning, neutralization techniques, student misconduct

## Abstract

Academic dishonesty is a widespread form of student misconduct in higher education, driven by cognitive rationalizations that enable unethical behaviors while preserving a sense of moral reasoning. This study examines the relationship between academic dishonesty and neutralization techniques, in an Italian university sample. We focus on how students justify cheating through rationalization processes. Utilizing a cross-sectional survey, 205 Italian university students recruited through Prolific completed the Neutralization Techniques Scale and the Academic Dishonesty Scale. Demographic data were also collected, and analyses were conducted using SAS. Results demonstrated a positive correlation between neutralization techniques and academic dishonesty (*r* = 0.63; *p* < 0.001). Among neutralization strategies, denial of responsibility exhibited the strongest correlation with dishonest behavior (*r* = 0.57; *p* < 0.001), followed by condemnation of the condemners (*r* = 0.56; *p* < 0.0001), appeal to higher loyalties (*r* = 0.45; *p* < 0.0001), and denial of injury (*r* = 0.37; *p* < 0.0001). These findings underscore the role neutralization techniques play in enabling academic dishonesty by allowing students to justify unethical actions and mitigating feelings of guilt or internal conflict. Replicating this pattern in an Italian sample highlights the importance of addressing these cognitive justifications through targeted interventions such as training programs that challenge common rationalizations and clarify norms around academic integrity and AI use. While the cross-sectional design, reliance on self-report measures, and use of an online convenience sample limit causal inference and generalizability, but nonetheless provide a robust basis for advancing theory-driven interventions targeting academic dishonesty.

## 1 Introduction

Academic dishonesty, including academic fraud, cheating, and related misconduct, is a widespread issue in higher education worldwide (Chernoff et al., 2025). Although no single definition is universally accepted (Bleeker, 2008), the term generally refers to behaviors aimed at gaining an unfair or unearned advantage in academic contexts (Hamid et al., 2017; Hughes and McCabe, 2006; Miles et al., 2022; Recommendation CM/REC, 2022; East and Donnelly, 2012). Such behaviors undermine academic integrity, which rests on values of honesty, trust, fairness, respect, responsibility, and courage (East and Donnelly, 2012; Finn and Frone, 2004; Nesterova, 2021; Sozon et al., 2024). Institutions globally monitor the prevalence and consequences of academic dishonesty, underscoring the need for effective interventions (Beck, 2014).

Academic dishonesty encompasses a range of behaviors. Pavela distinguishes four main forms—cheating, fabrication, facilitation, and plagiarism (Pavela, 1997)—while Whitley differentiates between total cheating, cheating on examinations, cheating on homework, and plagiarism (Whitley, 1998). Across classifications, common elements include deception, intentional rule violations, and disregard for academic norms. Although some of these behaviors could theoretically lead to legal sanctions, institutions typically address them internally, and formal penalties are rarely pursued (Kelley and Kimberly Bonner, 2005; De Maio et al., 2019). As a result, many cases go unreported or undetected, suggesting that the true prevalence is likely underestimated (Chirikov et al., 2020; Burke and Sanney, 2018).

Recent concerns about ethical conduct in academia have been amplified by high-profile cases and growing scholarly attention to academic integrity (Maral, 2024; Kisamore et al., 2007). Empirical studies across countries (e.g., Sweden, Poland, USA) consistently show high levels of dishonest behavior (Trost, 2009; Lupton et al., 2000; Foltýnek and Glendinning, 2015; McCabe et al., 2001; Vandehey et al., 2007). The expansion of online learning and generative AI tools has introduced additional vulnerabilities: students show favorable or ambivalent attitudes toward AI systems such as ChatGPT (Ofem et al., 2025; Lo, 2023; Cotton et al., 2024), and online examination environments during the COVID-19 pandemic have been associated with increased cheating (Newton et al., 2023).

Multiple individual and contextual factors contribute to dishonest behavior such as language barriers, academic stress, and unfamiliarity with academic conventions (McNair and Oye, 2018; Cronan et al., 2018; Doss et al., 2016). Students frequently report uncertainty about what constitutes cheating, especially in relation to collaboration and citation practices (Cronan et al., 2018), and many feel unprepared for university-level academic writing (McNair and Oye, 2018). Perceptions that “everyone cheats” or that dishonesty is necessary for success can normalize such behaviors (Miles et al., 2022; Grenness, 2023). Moral reasoning, self-esteem, and parenting or teaching styles also influence attitudes toward misconduct (Miles et al., 2022).

Among psychological factors or traits such as impulsivity, machiavellianism, narcissism, moral disengagement, and self-efficacy, neutralization techniques appear particularly relevant (Meng et al., 2014; Lue and Stiles, 2022; Lee et al., 2020; Haines et al., 1986; Faulkner, 2012; Barbaranelli et al., 2018). Introduced by Sykes and Matza to explain juvenile delinquency, neutralization theory remains broadly used in criminology and deviance research (Lee et al., 2020; Sykes and Matza, 2017). It proposes that individuals justify norm-violating behavior by weakening its conflict with personal moral standards, thereby reducing cognitive dissonance and guilt and preserving a positive self-image (Sykes and Matza, 2017; Harmon-Jones and Mills, 2019; Miller et al., 2015).

Sykes and Matza identified five techniques: denial of responsibility, denial of injury, denial of the victim, condemnation of the condemners, and appeal to higher loyalties (Sykes and Matza, 2017). Among these prior research shows that denial of responsibility, denial of injury, and condemnation of the condemners are especially common among university students (Lee et al., 2020; Poltorak, 1995; Olafson et al., 2013; Curasi, 2013; Brent and Atkisson, 2011; Beasley, 2014; Awdry and Groves, 2023).

International studies confirm both the prevalence of academic dishonesty and the widespread use of neutralization strategies. Research from the USA (Stephens et al., 2007; Smith et al., 2021; Smith et al., 2023), Australia (Eriksson and McGee, 2015), China (Tsui and Ngo, 2016), the UAE (Al Serhan et al., 2022), Korea (Park et al., 2013), Malaysia (Ismail and Yussof, 2016), Indonesia (Sasongko et al., 2019), India (Ali and Masood, 2019), Pakistan (Ellahi et al., 2013), Russia (Poltorak, 1995), Turkey (Bicer, 2020), Jordan (Al Shbail et al., 2022), Uganda (Nkundabanyanga et al., 2014), and Portugal (Dias-Oliveira et al., 2024) shows that students frequently rationalize cheating by minimizing harm, externalizing blame, or emphasizing structural pressures.

Despite extensive international evidence, research on neutralization techniques in Italy remains limited. This gap is theoretically consequential, as it prevents a nuanced understanding of how Italian students cognitively reconcile academic misconduct with broader moral and civic norms. This issue is particularly salient in fields associated with positions of high trust (e.g., healthcare, law or education) (Barbaranelli et al., 2018; Nonis and Swift, 2001; Harding et al., 2004; Gabriel Guerrero-Dib et al., 2020). Accordingly, examining neutralization techniques in an Italian sample allows for an assessment of how culturally embedded narratives about responsibility, merit, and systemic constraint shape the moral psychology of academic dishonesty in a context where the stakes of ethical formation are particularly pronounced.

Against this background, the present study examines, for the first time, the relationship between academic dishonesty and neutralization techniques among Italian university students. We investigate how students justify dishonest behaviors and how these rationalizations relate to self-reported misconduct. Using validated instruments and a correlational design, we hypothesized a strong positive association between neutralization techniques and academic dishonesty. We also explored how specific neutralization strategies relate to different forms of cheating. Details of the preregistered analysis plan and materials are provided in the Method section.

## 2 Methods

### 2.1 Participants

Two-hundred and five Italian residents currently enrolled in a university program participated in the study (97 females, 97 males, 11 non-binary/other, *M*age = 24.67, *SD*age = 4.18). Most participants were early in their university studies (Median Years Completed = 1, IQR = 1–2). The sample was predominantly white (≈92%) and largely non-religious (65.9% reported no religious affiliation). Participants were distributed across diverse fields of study, with the largest groups in Informatics, Engineering, Psychology, and Medicine. Self-reported English proficiency was high (*M* = 4.60/5, SD = 0.54), and subjective socio-economic status was moderate (*M* = 5.40/9, SD = 1.41). Participants were recruited through Prolific’s online platform, which allowed us to target residents of Italy currently enrolled in university programs. Eligibility criteria included being at least 18 years old and currently attending an undergraduate or graduate university program. Quotas for gender and age were not imposed, but the platform enabled access to a relatively diverse student population across fields of study and geographic regions. While the sample provides a reasonable cross-section of Italian university students, the findings should be interpreted with caution and are not automatically generalizable to all European undergraduates.

### 2.2 Materials and procedure

After providing consent, participants were given a brief overview of the study objectives before completing the key measures. The survey began with the assessment of neutralization techniques, followed by an academic dishonesty scale. Demographic information, including age, gender, ethnicity, years of university completed, field of study, importance of religion, type of religion, political ideology, subjective socio-economic status, and self-reported familiarity with English, was collected at the end.

### 2.3 Aim of the study and sample size

The primary aim of this pre-registered correlational study was to examine the association between neutralization techniques and the frequency of academic cheating behaviors among Italian university students, and to compare this pattern with those reported in international samples (Lee et al., 2020; Haines et al., 1986; Curasi, 2013; Awdry and Groves, 2023; Dias-Oliveira et al., 2024). In addition to this overall association, we explored the relationships between specific neutralization techniques (denial of responsibility, denial of injury, condemnation of the condemners, appeal to higher loyalties) and distinct dimensions of academic dishonesty (e.g., cheating in exams, plagiarism, falsification).

Because validated Italian versions of the relevant instruments were not available, all scales were administered in English. To ensure comprehensibility, a preliminary pre-test was conducted on a separate sample of 25 participants prior to the main data collection. Participants reported relatively high levels of English proficiency (*M* = 4.12, SD = 0.73), and no major comprehension issues emerged. Exploratory analyses further suggested that English proficiency was not significantly associated with the main study variables, although the limited sample size and restricted variability warrant cautious interpretation. We determined the sample size *a priori* to ensure adequate statistical power: power analyses indicated that a sample of at least 200 participants would provide ≥ 85% power to detect medium-sized effects (r ≈ 0.30) at *α* = 0.05 (two-tailed). On this basis, we recruited 205 Italian university students via Prolific.

The study was conducted in accordance with the ethical standards of Franklin University Switzerland and Hochschule für Wirtschaft Zürich and received approval from both institutions’ Institutional Review Boards. The study was pre-registered, and all research materials and anonymized data are publicly available on the Open Science Framework.^1^ The complete pre-registration is accessible at https://aspredicted.org/644j-bwnc.pdf.

### 2.4 Neutralization techniques

Neutralization strategies were measured using a modified version of the 11-item Neutralization Scale by Haines et al. (1986), reduced to 9 items for relevance to academic contexts (for a similar approach see Curasi (2013)). Participants rated their agreement with statements representing neutralization strategies on a 5-point scale ranging from “strongly disagree” (Al Serhan et al., 2022) to “strongly agree” (Aronson and Aronson, 2018). Following McCabe (1992) and Curasi (2013), we grouped the individual questions into four summary variables representing the five techniques of neutralization. Specifically, we combined denial of the victim and condemnation of the condemners into a single summary variable, because these factors appear largely indistinguishable in academic settings (Haines et al., 1986; Curasi, 2013).

The scale demonstrated good internal reliability (*α* = 0.85). We computed a composite neutralization index as the average of the 9 items. In addition, we computed separate scores for each of the four resulting neutralization techniques, all of which showed acceptable internal consistency.

### 2.5 Academic cheating

Academic cheating was measured using the 23-item Academic Dishonesty Scale (ADS) developed by Bashir and Bala (2018). The ADS assesses the frequency of dishonest academic behaviors across six dimensions: cheating in examinations, plagiarism, seeking outside help, prior cheating, falsification, and lying about academic assignments. Response options ranged from “I would never do it” (Chernoff et al., 2025) to “I would do it regularly” (Miles et al., 2022). The scale demonstrated high internal reliability in the present sample (α = 0.87), and each factor showed acceptable internal consistency.

## 3 Results

We first examined the relationship between neutralization techniques and academic dishonesty using Pearson correlation coefficients. As predicted, we found a significant positive correlation between the composite measure of neutralization (*M* = 2.72, *SD* = 0.84) and the composite measure of academic cheating (*M* = 1.63, *SD* = 0.43), *r*(203) = 0.63, *p* < 0.001. This suggests that higher levels of neutralization were associated with a greater frequency of cheating behaviors.

### 3.1 Correlations by neutralization factors

Each specific neutralization technique exhibited varying degrees of relationship with our composite index. Denial of responsibility (*M* = 2.47, *SD* = 0.94) demonstrated the strongest relationship with academic dishonesty, *r*(203) = 0.57, *p* < 0.0001, highlighting the tendency of students to justify dishonest behavior by externalizing blame. Denial of injury (*M* = 2.36, *SD* = 1.13) showed a moderate correlation with academic cheating, *r*(203) = 0.37, *p* < 0.0001, suggesting that minimizing the perceived harm of one’s actions might contribute to the tendency to engage in dishonest behavior. Condemnation of the condemners (*M* = 2.74, *SD =* 1.00) also showed a strong positive association with academic dishonesty, *r*(203) = 0.56, *p <* 0.0001, indicating that discrediting the authority enforcing rules facilitated academic dishonesty. Finally, appeal to higher loyalties (*M =* 3.27, *SD* = 1.14) was moderately correlated with academic cheating, *r*(205) = 0.45, *p* < 0.0001, implying that prioritizing loyalty to a group or causes over personal integrity can lead to dishonest behaviors.

### 3.2 Correlations between neutralization techniques and cheating dimensions

We further explored the relationship between neutralization factors and individual dimensions of academic cheating (see Table 1). Results indicated varied influences on specific types of academic dishonesty. Denial of responsibility was particularly prominent in rationalizing actions related to cheating on exams and seeking outside help, suggesting that students often deflect accountability when rules are perceived as overly strict or external pressures are high. Condemnation of the condemners also featured strongly in these contexts, reflecting challenges to institutional authority as a justification for dishonesty.

**Table 1:** Correlations between neutralization techniques and dimensions of academic dishonesty.

NT	ACI	CE	PL	OH	PC	FA	LAE
DOR	0.58***	0.52***	0.22**	0.50***	0.55***	0.22**	0.33***
DOI	0.38***	0.40***	0.15*	0.32***	0.33***	0.14*	0.17*
COC	0.56***	0.48***	0.37***	0.44***	0.46***	0.17*	0.32***
AHL	0.45***	0.38***	0.23**	0.35***	0.34***	0.15*	0.38***

***p < 0.001, **p < 0.01, *p < 0.05. NT, Neutralization technique; ACI, Academic cheating index; CE, Cheating in exams; PL, Plagiarism; OH, Seeking outside help; PC, Prior cheating; FA, Falsification; LAE, Lying about exams; DOR, Denial of responsibility; DOI, Denial of injury; COC, Condemnation of the condemners; AHL, Appeal to higher loyalties.

Plagiarism was more closely associated with condemnation of the condemners and appeal to higher loyalties, implying that students who discredit academic rules or prioritize loyalty to others may rationalize misappropriating others’ work. Additionally, falsification and lying about assignments were often justified by appeal to higher loyalties, indicating that social bonds or obligations frequently override consideration for academic integrity.

### 3.3 Exploratory analysis

We conducted an exploratory multiple regression analysis to predict academic dishonesty from the composite score of neutralization techniques while controlling for age, gender, perceived importance of religion, years of university completed, political affiliation, subjective socio-economic status, and English proficiency. The model significantly predicted academic dishonesty, *F*(8, 196) = 17.33, *p* < 0.001, explaining approximately 41.4% of the variance (*R*^2^ = 0.414, adjusted *R*^2^ = 0.390). Neutralization techniques remained a significant positive predictor of academic cheating (*β* = 0.323, *SE* = 0.029, *t*(196) = 11.09, *p* < 0.001), indicating that higher endorsement of neutralization techniques is associated with greater academic dishonesty. None of the demographic or background variables significantly contributed (all *p*s > 0.05). Given the possibility that the relationship between neutralization and academic cheating may becomplex, we conducted a multiple regression predicting neutralization techniques using academic cheating and the same control variables. The model was also significant, *F*(8, 196) = 19.25, *p* < 0.001, explaining approximately 44% of the variance (*R*^2^ = 0.440, adjusted *R*^2^ = 0.417). Academic cheating remained a significant predictor of neutralization (*β* = 1.195, *SE* = 0.107, *t*(196) = 11.09, *p* < 0.001), with years of university completed as the only significant positive predictor (*p* < 0.05). These findings further support a robust association between neutralization techniques and academic dishonesty and are consistent with the possibility of a reciprocal relationship, although the cross-sectional design does not allow any inference regarding causal direction.

## 4 Discussion

Our findings indicate a strong association between neutralization techniques and academic dishonesty. Students who more strongly endorsed neutralization strategies reported higher levels of cheating, and the reverse model predicting neutralizations from cheating was similarly robust. These results align with international evidence showing that rationalizing unethical behavior is closely associated with academic misconduct (Meng et al., 2014; Lee et al., 2020; Barbaranelli et al., 2018; Curasi, 2013; Awdry and Groves, 2023; Stephens et al., 2007; Smith et al., 2021; Smith et al., 2023; Bicer, 2020; Dias-Oliveira et al., 2024). The absence of significant effects for demographic variables (except for years of university completed in the reverse model) suggests that this association may reflect a general psychological mechanism rather than systematic differences. However, the cross-sectional design precludes any inference regarding causal direction.

This mechanism can be interpreted through theories of moral disengagement, cognitive dissonance, and self-concept maintenance. Bandura’s work on moral disengagement describes how individuals justify harmful actions by minimizing agency or distorting consequences to reduce self-condemnation (Bandura, 2016; Bandura, 1999). In our data, the prominence of denial of responsibility and condemnation of the condemners aligns with these processes, allowing students to maintain a positive self-view while violating academic norms. Cognitive dissonance theory similarly suggests that behavior inconsistent with personal moral standards produces discomfort, prompting individuals to adjust beliefs or generate justifications (Harmon-Jones and Mills, 2019; Miller et al., 2015). From this perspective, academic dishonesty could lead students to adopt neutralizations *post hoc*, normalizing misconduct and increasing its likelihood in the future (Rettinger and Kramer, 2009; Mazar et al., 2008).

The theory of self-concept maintenance provides a complementary explanation: individuals tend to operate in a “grey zone,” cheating enough to gain benefits while preserving a self-image of honesty (Mazar et al., 2008). Neutralization strategies help maintain this balance by supporting justifications such as “everyone does it”, “no one is harmed”, or “the rules are unfair”. The moderate-to-strong correlations between specific neutralization factors and different dimensions of cheating (e.g., exam cheating, seeking outside help, plagiarism, lying about exams) support the idea that neutralizations function as flexible, situationally tailored justifications, even though the underlying directionality of these relationships cannot be determined from our data.

The Italian sample appears broadly similar to those from other cultural contexts, particularly in the centrality of denial of responsibility and condemnation of the (Lee et al., 2020; Olafson et al., 2013; Curasi, 2013; Brent and Atkisson, 2011; Beasley, 2014; Awdry and Groves, 2023; Eriksson and McGee, 2015; Dias-Oliveira et al., 2024). Students often reframed misconduct as a response to institutional demands or perceived injustice, a pattern consistent across disciplines and countries (Lue and Stiles, 2022; Eriksson and McGee, 2015; Ismail and Yussof, 2016; Bicer, 2020; Dias-Oliveira et al., 2024; Makarova, 2019; LaBeff et al., 1990; Aplin-Snider et al., 2021).

However, denial of injury played a comparatively smaller role, despite its prominence in other studies (Lee et al., 2020; Poltorak, 1995; Olafson et al., 2013; Brent and Atkisson, 2011; Dias-Oliveira et al., 2024). One interpretation is that Italian students may still view cheating as morally problematic even when direct harm is minimized, relying more on externalization of responsibility. Appeal to higher loyalties showed moderate associations, particularly with plagiarism and falsification, suggesting that loyalty-based obligations (e.g., helping friends or family) can override formal integrity norms, a finding consistent with prior work (Curasi, 2013; Dias-Oliveira et al., 2024).

Cultural frameworks such as Hofstede’s dimensions may help contextualize these patterns (Hofstede, 2001). Italy’s relatively high uncertainty avoidance and moderate-to-high power distance may contribute to perceptions of institutional rules as rigid or disconnected from students’ realities, increasing reliance on rationalizations targeting authority or structural constraints. In addition, Italian universities often operate in relatively hierarchical academic settings, where professors are viewed as central figures in evaluating performance and enforcing rules (Barbaranelli et al., 2018; Curasi, 2013; Marcarelli and Mancini, 2022). Coursework and assessment practices are frequently formalized, and students may perceive strong expectations to comply with established norms (Barbaranelli et al., 2018; Curasi, 2013; Marcarelli and Mancini, 2022). Strong family and peer bonds may also support higher-loyalty appeals. These interpretations remain speculative, as cultural dimensions were not directly measured in the present study and should therefore be interpreted with caution and tested in future research (Miles et al., 2022; Eriksson and McGee, 2015; Gabriel Guerrero-Dib et al., 2020; Makarova, 2019).

Finally, our exploratory analyses echo prior work examining causal pathways. Studies indicate that rationalizing attitudes both predict and emerge from cheating behavior (Rettinger and Kramer, 2009), and decision-tree analyses identify rationalization as a key factor distinguishing cheaters from non-cheaters (Bicer, 2020). Viewed alongside the present findings, this literature is consistent with the possibility that neutralizations and misconduct may be related over time, although the cross-sectional design does not allow any inference regarding temporal or causal ordering. Establishing the temporal ordering of these processes will require longitudinal or experimental designs, which we identify as priorities for future research.

### 4.1 Practical implications

Our findings underscore the need for systemic interventions that directly address the cognitive processes enabling academic dishonesty. Educational strategies should target neutralization techniques, supporting students in recognizing and critically evaluating common rationalizations such as “everyone does it” or “the exam was unfair”. Promoting core values such as honesty, trust, and fairness, together with clear definitions of academic integrity, remains essential (Miles et al., 2022; East and Donnelly, 2012; Nesterova, 2021; McIntire et al., 2024; Mc Gowan, 2016; Macdonalda and Carrollb, 2006; Davis et al., 1992; Belter and du Prã©, 2009). Transparent communication about plagiarism, acceptable collaboration, and the legitimate use of AI tools can further reduce ambiguity and limit opportunities for rationalization (Cronan et al., 2018; McIntire et al., 2024; Belter and du Prã©, 2009; Sotiriadou et al., 2020).

In the Italian context, coherent and accessible integrity policies are particularly important. European evidence suggests that many undergraduates have only a general understanding of integrity principles and often encounter “grey zones” created by unclear or inconsistently applied guidelines (Goddiksen et al., 2024). This appears especially relevant in Italy, where institutional communication on ethical norms is frequently fragmented, leaving students uncertain about what constitutes misconduct or acceptable cooperation.

A recent meta-analysis indicates that integrity training is most effective when students actively engage with ethically challenging scenarios and reflect on their own responses (Katsarov et al., 2021). Tools such as the “Integrity Games” platform illustrate how interactive, scenario-based training can complement traditional instruction (Goddiksen et al., 2024). Integrating similar approaches into Italian universities may foster deeper ethical reflection and help students anticipate rationalizations before they arise.

Universities should also refine assessment strategies and organizational practices to reduce incentives for cheating. Effective measures include varied and authentic assessment formats, oral or competency-based evaluations, and clear expectations regarding AI use (Newton et al., 2023; Nkundabanyanga et al., 2014; McIntire et al., 2024; Mc Gowan, 2016; Macdonalda and Carrollb, 2006; Davis et al., 1992; Sattler et al., 2017; Whisenhunt et al., 2022; Nguyen et al., 2020; Morris, 2018). Given the role of appeal to higher loyalties, strategies that limit opportunities and clarify the boundary between legitimate collaboration and cheating are particularly relevant.

Embedding peer-accountability mechanisms may also help counter rationalizations grounded in social obligations, while avoiding strictly punitive framing that may encourage externalization of blame (Bandura, 2016; Bandura, 1999; Mazar et al., 2008).

Consistent and transparent regulation is equally essential. Inconsistently applied sanctions can create a permissive climate in which cheating is perceived as low-risk (Kelley and Kimberly Bonner, 2005; De Maio et al., 2019; Chirikov et al., 2020; Sattler et al., 2017). Clear codes of conduct, accessible reporting procedures, and proportionate sanctions emphasizing both fairness and educational value are therefore crucial (Recommendation CM/REC, 2022; Burke and Sanney, 2018; Harding et al., 2004; Birks et al., 2020; McCabe and Pavela, 2004).

Finally, the positive association between years of university completed and endorsement of neutralization techniques underscores the importance of early intervention. Patterns of dishonest behavior and associated rationalizations may consolidate over time and extend into professional contexts, where the consequences can be substantial (Beasley, 2014; Nonis and Swift, 2001; Gabriel Guerrero-Dib et al., 2020; Hofstede, 2001; Nonis and Swift, 2015). Integrity training should therefore begin early and be reinforced throughout the curriculum, particularly in programs that lead to professions of public trust (e.g., medicine, nursing, law, engineering).

### 4.2 Study limitations and future research

This study has several limitations. First, its cross-sectional design precludes causal inferences. Longitudinal research is needed to determine whether neutralizations predict later cheating, whether cheating increases rationalization, or whether both processes mutually reinforce one another over time (Meng et al., 2014; Lee et al., 2020; Bicer, 2020; Dias-Oliveira et al., 2024; Rettinger and Kramer, 2009). Such designs would allow testing the temporal ordering of these processes. Experimental interventions that explicitly target neutralization strategies (e.g., training that challenges common rationalizations) could further clarify whether modifying these cognitions reduces dishonest behavior.

Future studies should also examine the psychological and social mechanisms that mediate or moderate this link. Factors such as academic stress, perceived institutional fairness, group norms, peer influence, and attitudes toward authority may shape how neutralizations arise and whether they translate into action (Miles et al., 2022; Grenness, 2023; Eriksson and McGee, 2015; Makarova, 2019; Morris, 2018). Investigating these variables in larger and more diverse Italian samples would help refine theoretical models and support mechanism-based interventions.

Second, the reliance on self-report measures introduces the risk of social desirability and recall biases. Students may under-report dishonest behavior or present their rationalizations in a more socially acceptable light, potentially inflating justification tendencies, especially for stigmatized actions such as plagiarism or exam cheating (Ofem et al., 2025; Doss et al., 2016; Stephens et al., 2007; Ellahi et al., 2013; Sattler et al., 2017). Future research could triangulate self-reported data with behavioral tasks, experimental paradigms, or anonymized institutional indicators (e.g., plagiarism-detection reports), while attending to ethical and privacy considerations.

Third, the sample was recruited exclusively through Prolific, which may limit generalisability. Self-selection into online platforms and uneven socio-demographic representation can affect estimates of both dishonesty and neutralization (McNair and Oye, 2018; Nkundabanyanga et al., 2014; Nguyen et al., 2020). Broader recruitment strategies, including multiple universities, regions, and study programs, would enhance external validity and allow more nuanced analyses of demographic and contextual moderators.

Although participants reported familiarity with the language, and a preliminary pre-test did not reveal major comprehension issues, subtle comprehension differences may have influenced responses, particularly on sensitive items related to cheating and ethical justification. Future research should prioritize developing and validating Italian versions of the Neutralization Scale and the Academic Dishonesty Scale, following best practices for translation, cultural adaptation, and psychometric assessment (Meng et al., 2014; Haines et al., 1986; Bashir and Bala, 2018). Such work would also allow for more precise cross-cultural comparisons.

Finally, this study does not address in depth the risks posed by rapidly evolving AI technologies. Tools such as ChatGPT can enable undetectable plagiarism, automate assignment production, and provide real-time support during online exams (Sozon et al., 2024; Ofem et al., 2025; Lo, 2023; Cotton et al., 2024; Newton et al., 2023). These developments challenge conventional detection methods and raise new questions about what students consider “legitimate” technological assistance. Understanding how neutralization techniques interact with beliefs about AI (e.g., “using AI is just a study aid”) will be crucial for designing integrity interventions that remain effective in increasingly digital learning environments.

## 5 Conclusion

This study examined the relationship between neutralization techniques and academic dishonesty among Italian university students, providing the first systematic evidence on this topic in the national context. The results revealed a strong positive association between students’ endorsement of neutralization strategies and their self-reported engagement in academic cheating.

Among the different techniques, denial of responsibility and condemnation of the condemners emerged as the most consistent correlates of dishonest behavior, while denial of injury played a comparatively smaller role. These findings align with international evidence and suggest that students rely on context-dependent rationalizations to justify misconduct.

Although the cross-sectional design precludes conclusions about causal direction, the findings highlight neutralization techniques as meaningful psychological indicators of academic dishonesty. Addressing these rationalizations may support the development of more effective interventions, including integrity education that explicitly targets common justifications, clearer institutional guidelines, and assessment strategies that reduce opportunities for cheating.

Overall, the study underscores the importance of understanding academic dishonesty not only as a behavioral issue but also as a cognitive and moral process. Encouraging students to critically reflect on their own justifications may contribute to fostering a stronger culture of academic integrity.

## Data Availability

The datasets presented in this study can be found in online repositories. The names of the repository/repositories and accession number(s) can be found in the article/supplementary material.

## Footnotes

1
https://osf.io/xt3ab/?view_only=fc3e4ccaba1a4e67a8ebbd67c5e9da44
